# Robotic-assisted surgery versus open surgery in the treatment of rectal cancer: the current evidence

**DOI:** 10.1038/srep26981

**Published:** 2016-05-27

**Authors:** Guixiang Liao, Yan-Bing Li, Zhihong Zhao, Xianming Li, Haijun Deng, Gang Li

**Affiliations:** 1 Department of Radiation Oncology, Shenzhen People’s Hospital, Second Clinical Medicine College of Jinan University, China; 2 Department of Hepatobiliary and Pancreatic Surgery, Taihe Hospital, Hubei University of Medicine, Hubei, China; 3 Department of Nephrology, The Third Affiliated Hospital of Southern Medical University, Guangzhou, Guangdong, China; 4 Department of General Surgery, Nanfang Hospital of Southern Medical University, Guangzhou, Guangdong, China; 5 Department of Chemoradiation Oncology, The First Affiliated Hospital of Wenzhou Medical University, Wenzhou, China

## Abstract

The aim of this meta-analysis was to comprehensively compare the safety and efficacy of robotic-assisted rectal cancer surgery (RRCS) and open rectal cancer surgery (ORCS). Electronic database (PubMed, EMBASE, Web of Knowledge, and the Cochrane Library) searches were conducted for all relevant studies that compared the short-term and long-term outcomes between RRCS and ORCS. Odds ratios (ORs), mean differences, and hazard ratios were calculated. Seven studies involving 1074 patients with rectal cancer were identified for this meta-analysis. Compared with ORCS, RRCS is associated with a lower estimated blood loss (mean difference [MD]: −139.98, 95% confidence interval [CI]: −159.11 to −120.86; *P* < 0.00001), shorter hospital stay length (MD: −2.10, 95% CI: −3.47 to −0.73; *P* = 0.003), lower intraoperative transfusion requirements (OR: 0.52, 95% CI: 0.28 to 0.99, *P* = 0.05), shorter time to flatus passage (MD: −0.97, 95% CI = −1.06 to −0.88, *P* < 0.00001), and shorter time to resume a normal diet (MD: −1.71.95% CI = −3.31 to −0.12, *P* = 0.04). There were no significant differences in surgery-related complications, oncologic clearance, disease-free survival, and overall survival between the two groups. However, RRCS was associated with a longer operative time. RRCS is safe and effective.

Minimally invasive surgery is widely applied in many surgical fields. Robotic-assisted surgery is an advanced minimally invasive technique, has been applied in many branches of surgery (gynecologic^1^, urologic^2^, and gastrointestinal^3^), seems to achieve promising results, and has gained worldwide attention.

Robotic-assisted colorectal surgery was first reported in 2002^4^. Since then, a variety of reports regarding robotic-assisted rectal surgery have been published^5^,^6^,^7^,^8^,^9^. Robotic surgery is considered a good choice in the treatment of rectal cancer because this technique can overcome difficulties associated with the anatomy of the pelvis and has certain advantages^10^,^11^,^12^, including 3D imaging, dexterity and ambidextrous capability, lack of tremors, motion scaling, and a short learning curve^13^,^14^. Some reports have already indicated that robotic colorectal surgery has some benefits over conventional laparoscopic surgery based on observational comparative studies or randomized controlled trials^15^,^16^. However, the feasibility and safety of robotic-assisted rectal surgery compared with open rectal surgery in treating rectal cancer are not well elucidated. We conducted this meta-analysis to assess the safety and efficacy of robotic surgery versus open surgery in treating rectal cancer.

## Methods

### Search strategy

This meta-analysis was performed according to the Preferred Reporting Items for Systematic Reviews and Meta-Analyses (PRISMA) statement^17^. A comprehensive literature search was carried out by reviewers using the following electronic databases: PubMed, EMBASE, Web of Knowledge, and the Cochrane Library. The search was conducted in July 2014, and the language was restricted to English. The following search terms were used: robot or robotic or robot-assisted or da vinci or davinci, open, rectal or rectum or colorectal, and cancer or tumor or carcinoma.

The study inclusion criteria were as follows: (1) the study was a comparative study that compared the safety and efficiency between robotic rectal cancer surgery (RRCS) and open rectal cancer surgery (ORCS); (2) the study included quantitative outcome data (e.g., operative time, length of hospital stay, complications, pathological parameters, and survival outcomes); (3) if the same institution and/or authors reported several studies, only the study with the greatest patient population or the highest quality study was included in the analysis; and (4) the study was published in English. We excluded editorials, comments, meeting abstracts, review articles, and non-relevant topic studies.

### Data extraction

The primary relevant data from all the included studies were extracted by two reviewers (GXL, ZZ). The extracted data included the following: the basic characteristics of the study, including first author, year, and country of publication; the publication journal name; the basic patient characteristics, including age, sex, body mass index (BMI), American Society of Anesthesiologists (ASA) score, case number, and tumor stage; short-term outcomes, including intraoperative data, postoperative data, and oncologic clearance; and long-term outcomes, including disease-free survival and overall survival. All available data were extracted using standard data extraction by one reviewer and were checked by another reviewer.

### Quality assessment

Two reviewers (GL, YL) independently evaluated the quality of each included study using the modified Newcastle-Ottawa scale (available at http://www.ohri.ca/programs/clinical_epidemiology/oxford.asp), which is widely used for cohort study assessment. The quality assessment consisted of three major categories: patient selection, comparability of the RRCS and ORCS groups, and outcome assessment^18^. The details of this quality assessment are provided in Table S1. Any disagreement was resolved via discussion among the author group.

### Statistical analysis

This meta-analysis was performed using the Review Manager software (version 5.2, provided by the Cochrane Collaboration) and Stata software (version 11.0). Dichotomous variables were analyzed using odds ratios (ORs) with 95% confidence intervals (95% CIs). Survival outcomes were estimated using hazards ratios (HRs) and standard errors. Continuous variables were analyzed using mean differences (MDs) and 95% CIs. A fixed-effects model or a random-effects model was applied according to heterogeneity, which was evaluated by the I^2^ measure of inconsistency. Heterogeneity was present when the I^2^ statistic was greater than 50%, and a random-effects model was adopted. However, when the I^2^ statistic value was less than 50%, a fixed-effects model was used. Publication bias was evaluated by a funnel plot of postoperative complications and Egg’s and Begg’s tests. Sensitivity analysis was performed by excluding the low-quality studies. A *P* value less than 0.05 was considered to be significant.

## Results

### Literature search

**Figure 1:**
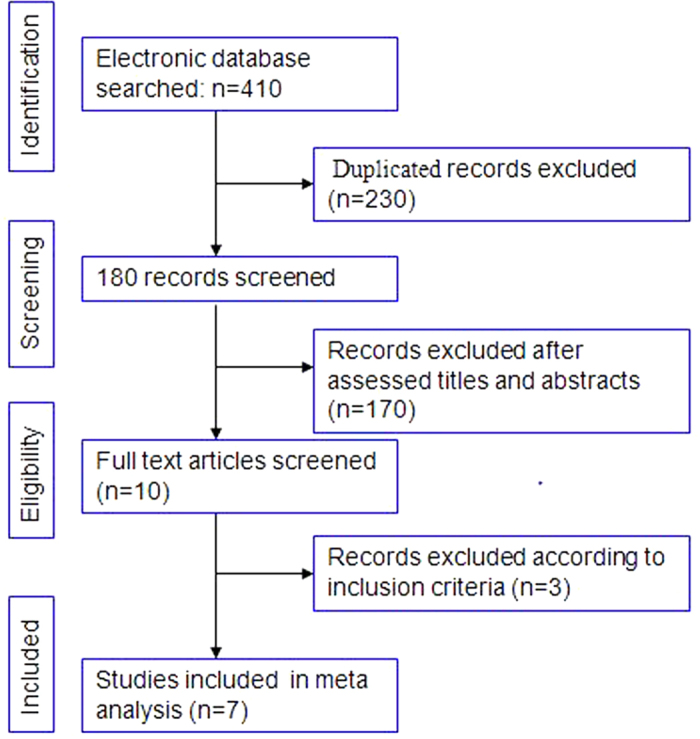
Flowchart of the literature search.

The PubMed, EMBASE, Web of Science, and Cochrane Library search identified a total of 277 studies. After excluding duplicates using Endnote software, 180 abstracts were carefully reviewed by two independent reviewers using a standard study selection form. After this process, the reviewers identified 10 studies for a comprehensive review. Three studies were excluded according to the inclusion criteria^19^,^20^,^21^, leaving 7 studies that were included in our analysis^22^,^23^,^24^,^25^,^26^,^27^,^28^. The study selection process is shown in Fig. 1. A total of 1074 patients with 498 (9.00%) cases of RRCS and 576 cases of ORCS were analyzed. Information on the studies and the participants is shown in Table 1. The quality assessment score of each included study is also provided in Table 1, and the details of each included study assessment are provided in Table S2.

### Meta-analysis

#### Intraoperative data

##### Operative times

**Figure 2:**
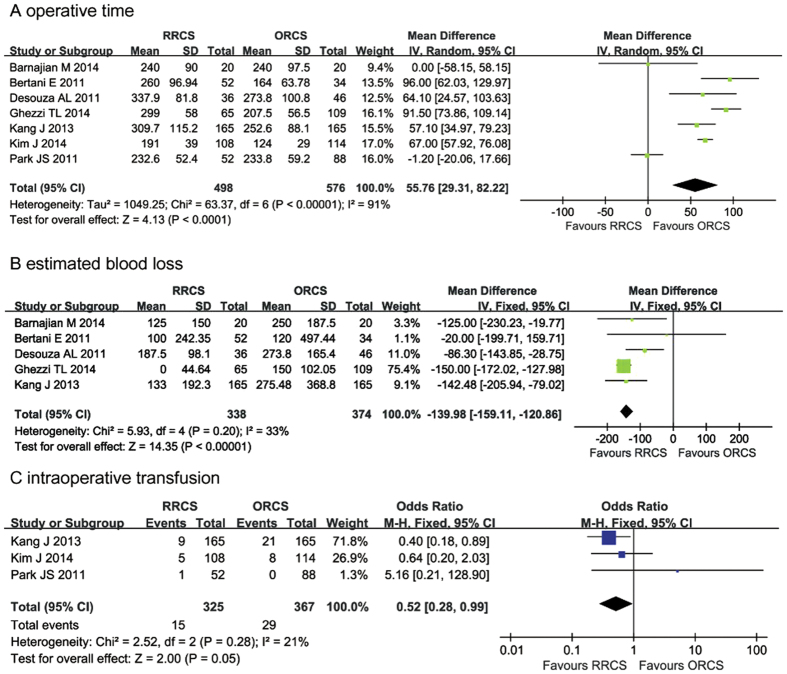
Meta-analysis of intraoperative data on robotic-assisted rectal cancer surgery versus open rectal cancer surgery. (**A**) operative time, (**B**) estimated blood loss, (**C**) intraoperative transfusion requirements.

The operative time was reported in all of the included studies^22^,^23^,^24^,^25^,^26^,^27^,^28^. The pooled data revealed that the operative time was significantly longer in the RRCS group compared with the ORCS group (MD = 55.76; 95% CI = 29.31–82.22; *P* < 0.0001), and the heterogeneity was high (*P* < 0.00001, I^2^ = 91%) (Fig. 2A).

##### Estimated blood losses

Five of the studies assessed intraoperative estimated blood loss (EBL)^22^,^23^,^24^,^25^,^26^. The EBL was significantly lower, by 139.98 ml, in the RRCS group compared with the ORCS group (MD: −139.98, 95% CI: −159.11 to −120.86; *P* < 0.00001). There was no significant heterogeneity (I^2^ = 33%, *P* = 0.20) (Fig. 2B).

#### Intraoperative transfusion

Intraoperative transfusion was mentioned in three studies^26^,^27^,^28^. Our results revealed that intraoperative transfusion requirements were reduced in the RRCS group compared with the ORCS group (OR: 0.52, 95% CI: 0.28–0.99, *P* = 0.05), and the analysis revealed significant heterogeneity (*P* = 0.28, I^2^ = 21%) (Fig. 2C).

#### Postoperative data

##### Overall postoperative complications

**Figure 3:**
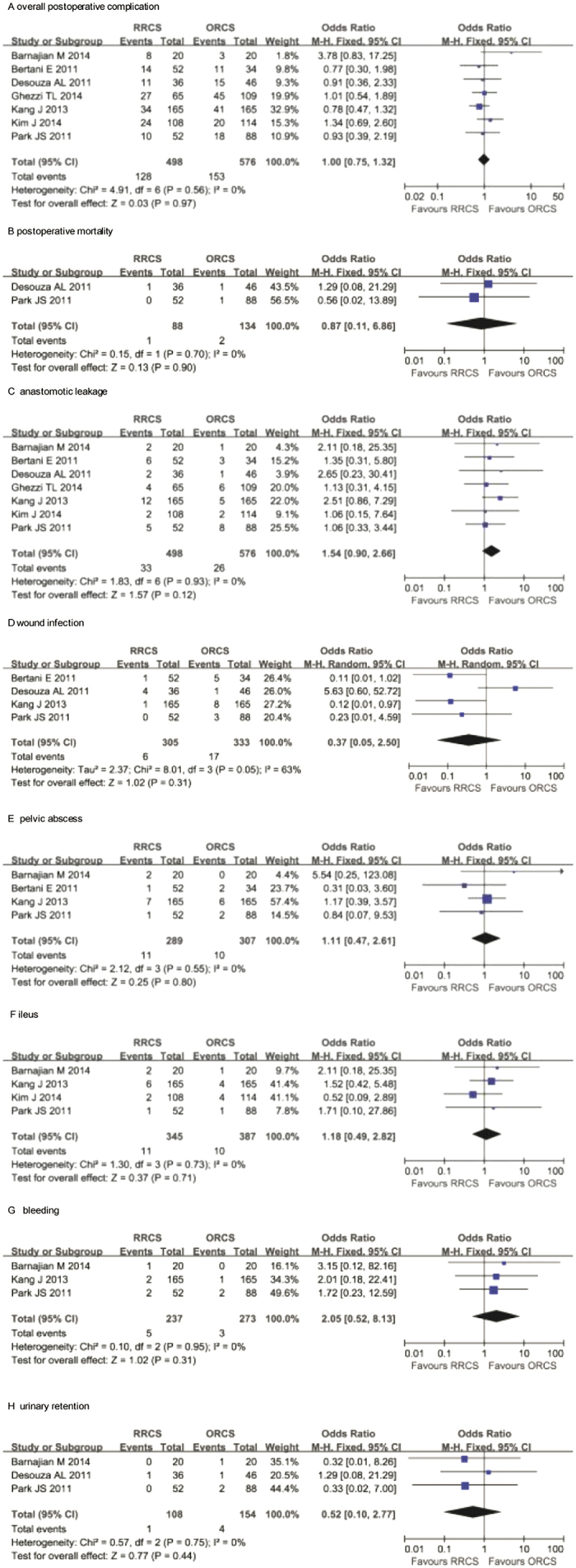
Meta-analysis of postoperative complications associated with robotic-assisted rectal cancer surgery versus open rectal cancer surgery. (**A**) overall postoperative complications, (**B**) postoperative mortality, (**C**) anastomotic leakage, (**D**) wound infection, (**E**) pelvic abscess, (**F**) ileus, (**G**) bleeding, (**H**) urinary retention.

All of the included studies mentioned this index^22^,^23^,^24^,^25^,^26^,^27^,^28^, and the overall postoperative complication rates were similar between studies. The pooled data showed that the total postoperative complications of the two groups were not significantly different (OR = 1.00, 95% CI: 0.75 −1.32, *P* = 0.97), and there was no evidence of heterogeneity (*P* = 0.56, I^2^ = 0) (Fig. 3A).

#### Postoperative mortality

Two studies mentioned postoperative mortality^24^,^28^. The pooled data analysis indicated that the mortality rate was not different between the two techniques (OR: 0.87, 95% CI: 0.11–6.86, *P* = 0.90), and there was no heterogeneity (*P* = 0.70, I^2^ = 0) (Fig. 3B).

#### Anastomotic leakage

Seven studies reported anastomotic leakage events^22^,^23^,^24^,^25^,^26^,^27^,^28^. The anastomotic leakage rate was 6.63% in the RRCS group and 4.51% in the ORCS group. The pooled data analysis revealed that this rate was not significantly different between the two groups (OR = 1.54, 95% CI = 0.90–2.66, *P* = 0.12), and there was no evidence of heterogeneity (*P* = 0.93, I^2^ = 0) (Fig. 3C).

#### Wound infection

Four studies reported the incidence of wound infection^23^,^24^,^26^,^28^. The combined data revealed that this parameter was not different between the two groups (OR = 0.37, 95% CI: 0.05–2.50, *P* = 0.31) (Fig. 3D).

#### Pelvic abscess

Four studies described the number of pelvic abscess events^23^,^24^,^26^,^28^. The pooled analysis indicated that there was no significant difference in this variable between the two approaches (OR = 1.11, 95% CI: 0.47–2.61, *P* = 0.80), and there was no heterogeneity (*P* = 0.55, I^2^ = 0) (Fig. 3E).

#### Ileus

Ileus events were reported in four studies^22^,^26^,^27^,^28^. The incidence of ileus in the RRCS group was 3.81%, compared with 3.26% in the ORCS group. The results showed no significant difference between the two groups (OR = 1.11, 95% CI: 0.47–2.61, *P* = 0.80), and there was no heterogeneity among the studies (Fig. 3F).

#### Bleeding

Three studies described bleeding^22^,^26^,^28^. The pooled data analysis revealed no significant difference in bleeding between the two techniques (OR: 2.05, 95% CI: 0.52–8.13, *P* = 0.31), and no significant heterogeneity existed among the studies (Fig. 3G).

#### Urinary retention

Three studies reported urinary retention^22^,^24^,^28^. The combined data indicated that urinary retention in the two groups was not significantly different (OR: 0.52, 95% CI: 0.10 to 2.77, *P* = 0.44), and there was no significant heterogeneity (Fig. 3H).

#### Length of hospital stay

**Figure 4:**
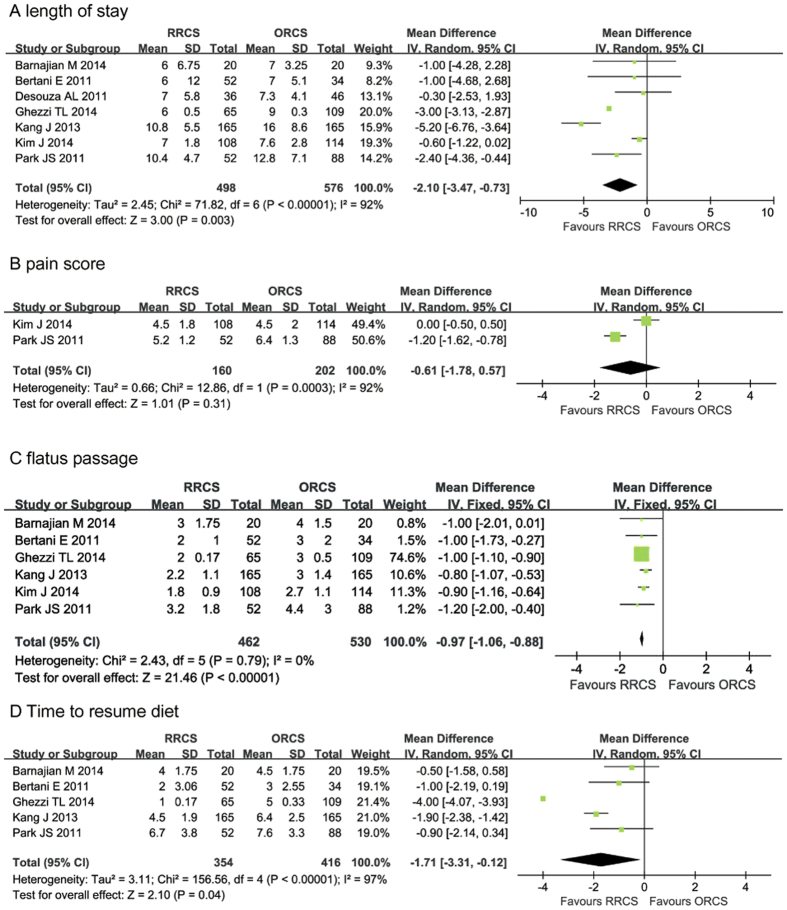
Meta-analysis of outcomes between robotic-assisted rectal cancer surgery and open rectal cancer surgery. (**A**) length of stay, (**B**) pain score, (**C**) flatus passage, (**D**) time to diet resumption.

All of the studies^22^,^23^,^24^,^25^,^26^,^27^,^28^ described length of hospital stay (LOS). The pooled data of the included studies showed that the LOS was significantly reduced in the RRCS group compared with the ORCS group (MD: −2.10, 95% CI: −3.47 to −0.73; *P* = 0.003). However, the heterogeneity was high (*P* < 0.00001, I^2^ = 92%) (Fig. 4A).

#### Pain score

Pain scores were reported in two studies^27^,^28^. The pooled data of the two studies showed no difference between the two approaches with respect to pain scores (MD: −0.61, 95% CI: −1.78 to 0.57, *P* = 0.31), and there was high heterogeneity (*P* = 0.003, I^2^ = 92%) (Fig. 4B).

#### Flatus passage

Six studies reported the time to flatus passage^22^,^23^,^25^,^26^,^27^,^28^. The combined data indicated that RRCS significantly reduced the time to first flatus passage by 0.97 days compared with the ORCS group (MD: −0.97, 95% CI = −1.06 to −0.88, *P* < 0.00001), without significant heterogeneity among the studies (*P* = 0.79, I^2^ = 0) (Fig. 4C).

#### Time to diet resumption

The time to normal diet resumption included the time to normal diet resumption and the time to resumption of a soft diet^22^,^23^,^25^,^26^,^28^. The combined analysis showed that the RRCS group had a significantly shorter time to diet resumption (MD: −1.71. 95% CI = −3.31 to −0.12, *P* = 0.04). However, there was high heterogeneity among the studies (*P* < 0.00001, I^2^ = 97%) (Fig. 4D).

#### Meta-analysis of the pathological details

**Figure 5:**
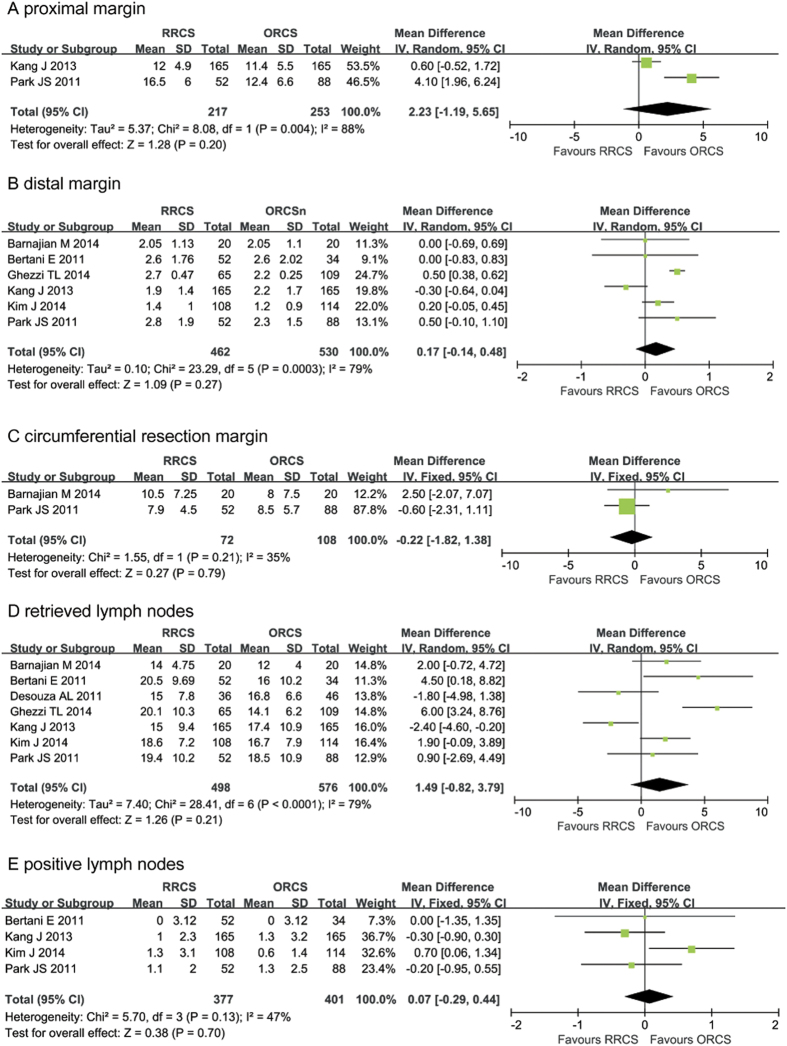
Meta-analysis of the pathological details between robotic-assisted rectal cancer surgery and open rectal cancer surgery. (**A**) proximal margin, (**B**) distal margin, (**C**) circumferential resection margin, (**D**) retrieved lymph nodes, (**E**) positive lymph nodes.

Kang *et al.*^26^ and Park *et al.*^28^ described proximal margin indices, and the combined data indicated no differences in this parameter (MD: 2.23, 95% CI: −1.19 to 5.65; *P* = 0.20, I^2^ = 88) (Fig. 5A). Six studies reported distal margins^22^,^23^,^25^,^26^,^27^,^28^, and there was no difference between the two groups in this parameter (MD: 0.17, 95% CI: −0.14 to 0.48; *P* = 0.27) (Fig. 5B), but there was significant heterogeneity (*P* = 0.0003, I^2^ = 79). Two studies mentioned circumferential resection margins^22^,^28^, and the pooled data revealed no significant differences (MD: −0.22, 95% CI: −1.82 to 1.38, *P* = 0.79) (Fig. 5C).

All the included studies reported the number of retrieved lymph nodes^22^,^23^,^24^,^25^,^26^,^27^,^28^. The combined data indicated that the two groups did not differ significantly in this parameter (MD: 1.49, 95% CI: −0.82 to 3.79; *P* = 0.21) (Fig. 5D), and there was significant heterogeneity (*P* < 0.00001, I^2^ = 79). Moreover, the pooled data showed no significant differences in the number of retrieved positive lymph nodes between the two groups (MD: 0.07, 95% CI: −0.29 to 0.44, *P* = 0.70) (Fig. 5E).

### Long-term outcomes

#### Disease-free survival (DFS)

**Figure 6:**
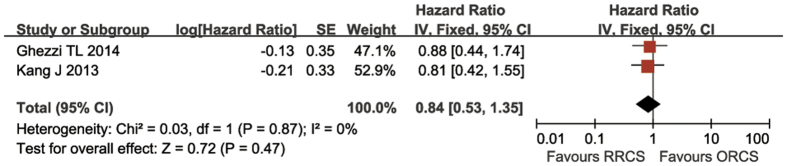
Meta-analysis of disease-free survival in the robotic-assisted rectal cancer surgery group compared with the open rectal cancer surgery group.

Two studies reported DFS outcomes^25^,^28^. Kang *et al.* reported that the 2-year DFS was 83.5% in the RRCS group and 79.7% in the ORCS group. Ghezzi *et al.*^25^ reported that the 5-year DFS was 73.2% and 69.5% in the RRCS and ORCS groups, respectively. The combined data indicated no differences in DFS between the two arms (HR: 0.84, 95% CI: 0.53–1.35, *P* = 0.47) and no heterogeneity (*P* = 0.87, I^2^ = 0) (Fig. 6).

#### Overall survival

Ghezzi *et al.*^25^ reported that the 5-year overall survival rate was higher in the RRCS group than the ORCS group, but this difference was not significant (85.0% vs. 76.1%). Future studies should be conducted to assess this index.

#### Publication bias

**Figure 7:**
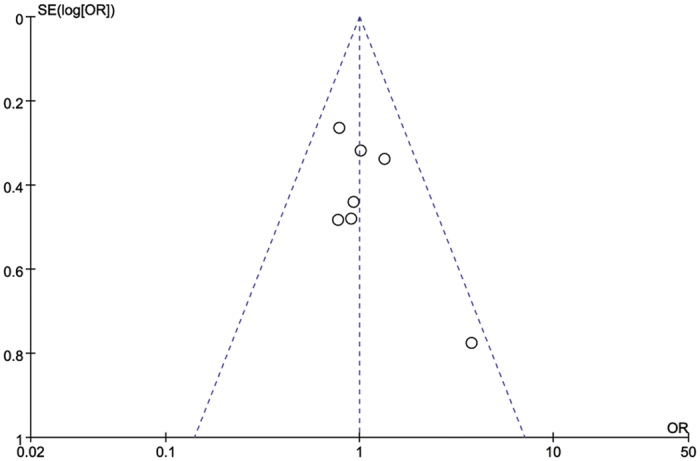
Funnel plot of overall postoperative complications associated with robotic-assisted rectal cancer surgery compared with open rectal cancer surgery.

A funnel plot analysis of all studies was performed in the meta-analysis of overall postoperative complications between RRCS and ORCS. Visually, all of the studies were within the limits of the 95% CIs (Fig. 7). Moreover, the statistical test indicated no evidence of publication bias (Egg’s test *P* = 0.174, Begg’s test *P* = 0.764).

#### Sensitivity analysis

A sensitivity analysis was performed by excluding the studies with a low quality score (Score ≤ 6)^27^,^28^. The results were not influenced, with the exception of the time to flatus passage. The results are listed in Table 2.

## Discussion

Several meta-analyses have evaluated the efficacy and safety of robotic-assisted surgery versus open surgery in gastric cancer^29^,^30^, pancreatic disease^31^, renal disease^32^, and bladder cancer^33^. All of these studies have indicated that robotic-assisted surgery is safe and effective. However, no meta-analysis has been conducted to evaluate robotic-assisted surgery compared with open surgery for rectal cancer.

This meta-analysis assessed the efficacy and safety of RRCS versus ORCS. This meta-analysis indicated that RRCS may provide certain benefits over ORCS. Compared with ORCS, RRCS is associated with a lower EBL (MD: −139.98, 95% CI: −159.11 to −120.86; *P* < 0.00001), shorter LOS (MD: −2.10, 95% CI: −3.47 to −0.73; *P* = 0.003), less intraoperative transfusion requirements (OR: 0.52, 95% CI: 0.28 to 0.99, *P* = 0.05), shorter time to flatus passage (MD: −0.97, 95% CI = −1.06 to −0.88, *P* < 0.00001), and shorter time to diet resumption (MD: −1.71.95% CI = −3.31 to −0.12, *P* = 0.04). There were no significant differences in overall postoperative complications, anastomotic leakage, pain scores, wound infection, ileus, pelvic abscess, bleeding, urinary retention, postoperative mortality, proximal margin, distal margin, circumferential resection margin, number of lymph nodes retrieved, number of positive lymph nodes retrieved, DFS, and overall survival between the two groups. However, the disadvantage of RRCS is that it was associated with a longer operative time (MD: −139.98, 95% CI: −159.11 to −120.86; *P* < 0.00001).

The combined data indicated that compared with the ORCS group, the EBL was significantly lower in the RRCS group. Due to a lower EBL, it is possible to suggest that RRCS may significantly reduce the probability of transfusion. Indeed, our meta-analysis suggested that the transfusion rate was significantly lower in the RRCS group compared with the ORCS group. Thus, reduced transfusion rates may prevent the recurrence of cancer^34^. Patients with cancer who receive more intraoperative blood transfusions are at greater risk for cancer recurrence, and the volume of transfused blood at surgery is an independent risk factor for cancer recurrence^35^. In addition, more EBL may indicate an unfavorable prognosis of patients with cancer^36^,^37^.

Another advantage of RRCS is its associated shorter LOS. The shorter LOS may be explained by the following rationale: RRCS is a minimally invasive surgery, and it may provide faster wound recovery and reduce postoperative pain. RRCS has a shorter time to flatus passage and a faster recovery to normal diet resumption.

The overall complication rate was similar between the two techniques (7% in the RRCS group and 8% in the ORCS group). This finding also indicated that RRCS is as safe and feasible as ORCS. The most frequently occurring events included anastomotic leakage, wound infection, ileus, and pelvic abscess. Our analysis indicated that the two groups had no differences in those regards.

Anastomotic leakage is a major surgical complication of gastrointestinal surgery^29^, and it represents one of the most dreaded complications of colorectal cancer. The mortality rate is high in patients with colorectal anastomotic leakage that required re-operation and accounts for almost 40% of the deaths after colorectal cancer surgery^29^. The anastomotic leakage rate was 6.49% (31/478) in the RRCS group and 4.5% (25/556) in the ORCS group, which was not a significant difference. The rate (6.49%) was consistent with previous reports for robotic surgery (1.8–12.1%)^5^,^38^,^39^ and was similar to the rate of 7% that was reported in open rectal cancer surgery based on a multicenter randomized controlled trial^40^.

The present study revealed no difference in oncologic clearance (proximal margin, circumferential resection margin, distal margin, and number of retrieved lymph nodes). Resection margins and number of retrieved lymph nodes have been regarded as quality indicators in rectal cancer surgery^41^ because the distal margin, circumferential resection margin, and number of retrieved lymph nodes were important parameters for the evaluation of prognosis in rectal cancer^42^,^43^,^44^,^45^. Several studies have indicated that RRCS can achieve good-quality performance.

The long-term follow-up was reported in two studies. The results indicated that there was no difference in DFS or overall survival between the two groups. Hara *et al.* reported that the 5-year overall survival and DFS rates were 88.6% and 76.6%, respectively, for patients diagnosed with stage III rectal cancer who had undergone robotic surgery^7^. The 3-year overall survival rate was 97%^46^.

Robotic rectal cancer surgery can achieve promising survival rates. Due to the limited number of studies, more studies are mandatory to establish the value of robotic surgery for rectal cancer in the future.

We should note the disadvantage of RRCS, namely, it requires a longer operative time compared with ORCS. This result is mainly attributed to the docking and preparation times associated with RRCS. A previous study reported that the median operative time for RRCS ranged from 240 to 310 min^46^. With increasing experience, the operative time would be reduced in robotic surgery^29^. It was reported that a surgical team requires approximately 30 cases to become comfortable and proficient with RRCS^28^.

The following limitations of this meta-analysis should be considered. First, the included studies were not randomized controlled trials; some studies were prospective studies, and some were retrospective studies. Thus, the studies may have been biased, and the results should be interpreted with caution. Second, as a novel technique, the cost-effectiveness of RRCS should be considered. Of the included studies, only that of Berten *et al.*^23^ reported the total cost, which was 11214€ for RRCS and 9858€ for ORCS; the combined data for this index were not available. However, a variety of studies have indicated that the cost of robotic colorectal surgery is higher than the cost of laparoscopic colorectal surgery^8^,^47^,^48^,^49^. The high capital and running costs of robotic systems have precluded their widespread use in many countries^50^. Third, the studies included patients with different basic characteristics and treatments, and these differences may have affected some of the results. Moreover, the surgeries in the included cases were carried out by different surgeons, and the different experiences and techniques of the surgeons may have affected some of the results^34^. Fourth, only two studies reported the survival outcomes after long-term follow-up; more studies are needed to assess the survival outcome as well as the recurrence events. Fifth, the studies included in this meta-analysis were limited to those published in the English language because the authors of the present study were not literate in other languages. Thus, studies published in English may have more frequently supported our hypotheses, and studies reported in other languages may have more frequently refuted our hypotheses.

In conclusion, this meta-analysis suggests that RRCS is safe and effective. RRCS was associated with reduced EBL, less intraoperative transfusion requirements, a shorter time to flatus passage, a shorter time to resumption of a normal diet, and reduced LOS. There were no significant differences in complication rates, oncologic clearance, and survival outcomes between the two groups. However, RRCS was associated with a longer operative time compared with ORCS. Future well-designed, larger, randomized controlled studies should be performed to assess the clinical and financial benefits and oncologic outcomes of RRCS to establish its role in the minimally invasive management of rectal cancer.

## Additional Information

**How to cite this article**: Liao, G. *et al.* Robotic-assisted surgery versus open surgery in the treatment of rectal cancer: the current evidence. *Sci. Rep.* **6**, 26981; doi: 10.1038/srep26981 (2016).

## Supplementary Material

Supplementary Tables

## Figures and Tables

**Table 1:** Basic characteristics of the included studies.

Author	Year	Country	Journal name	Study type	Group	N	Sex M/F	BMI	Age	ASA (1/2/3/4)	T Stage(0/1/2/3/4)	Operation type
Barnajian M^22^	2014	USA	Colorectal Dis	Retrospective case-matched	RRCSORCS	2020	12/812/8	22 (3.25)22 (3.25)	62 (9.5)61 (10)	0/4/16/00/4/16/0	NA	TME
Bertani E^23^	2011	Italy	Int J Colorectal Dis	prospective cohort comparative	RRCSORCS	5234	31/2120/14	24.8 (3.62)25.6 (3.85)	59.6 (11.6)63.2 (10.5)	49/3^a^28/6^a^	NA	TME
Desouza AL^24^	2011	USA	Dis Colon Rectum	Retrospective cohort comparative	RRCSORCS	3646	22/1425/21	27.4 (5.71)28.7 (6.58)	63.5 (11.5)63.7 (12.1)	27/9^a^31/15^a^	1/8/10/17/00/6/12/28/0	TME
Ghezzi TL^25^	2014	Brazil	Oncol	Prospective cohort comparative	RRCSORCS	65109	41/2461/48	24.7 (3.6)25.4 (3.6)	61.0 (11.8)61.1 (11.0)	12/49/4/016/63/29/1	10/5/17/27/615/10/38/42/4	TME
Kang J^26^	2013	Korea	Ann Surg Case-matched	Prospective	RRCSORCS	165165	104/61110/55	23.1 (2.8)23.0 (3.0)	61.2 (11.4)59.2 (11.0)	109/56/0/0125/40/0/0	31/42/87/5^b^31/48/78/8	TME
Kim JC^27^	2014	Korea	Surg Endosc	Retrospective cohort comparative	RRCSORCS	108114	64/4478/36	23.7 (2.7)23.2 (3)	57 ± 1161 ± 9	31/74/3/029/84/1/0	NA	AIR
Park JS^28^	2011	Korea	Surg Endosc	Retrospective cohort comparative	RRCSORCS	5288	28/2457/31	23.7 (2.4)23.3 (3)	57.3 (12.3)62.3 (10.4)	21/26/5/043/37/8/0	0/3/18/31/00/7/30/48/3	TME

^a^(1 + 2)/(3 + 4); TME: total mesorectal excision; AIR: abdominal intersphincteric resection.

^b^0 + 1/2/3/4.

**Table 2:** Sensitivity analyses excluding the low-quality studies.

Outcome	No. study	Patient		Effect measure	Analysis model	Effect and its 95% CI	*P* value	Heterogeneity	
		RRCS	ORCS					I^2^(%)	*P*
Operative time	5	338	374	MD	RE	68.47 (43.29,93.64)	<0.00001	71	0.0007
LOS	5	338	374	MD	RE	−2.52 (−4.08,−0.95)	0.002	75	0.003
Overall postoperative complications	5	338	374	OR	FE	0.93 (0.67,1.30)	0.68	0	0.42
Time to flatus passage	4	302	328	MD	FE	−0.98 (−1.07,−0.88)	0.00001	0	0.61
Time to resumption of a normal diet	4	302	328	MD	RE	−1.91 (−3.64,−0.17)	0.03	98	<0.00001
Anastomotic leakage	5	338	374	OR	FE	1.80 (0.94,3.46)	0.10	0	0.89
Wound infection	3	253	245	OR	RE	0.42 (0.03,5.05)	0.49	75	0.02
Pelvic abscess	3	237	219	OR	FE	1.16 (0.46,2.90)	0.75	4	0.35
Retrieved lymph nodes	5	338	374	MD	RE	1.55 (−1.88,4.99)	0.38	86	<0.00001
Distal margin	4	302	328	MD	RE	0.07 (−0.45,0.60)	0.78	86	<0.0001
Ileus	2	185	185	OR	FE	1.63 (0.52,5.09)	0.40	0	0.82
Urinary retention	2	56	66	OR	FE	0.67 (0.09,5.17)	0.70	0	0.52
Bleeding	2	185	185	OR	FE	2.38 (0.35,16.38)	0.38	0	0.83
Retrieved positive lymph nodes	2	217	199	MD	FE	−0.25 (−0.80,0.30)	0.37	0	0.69

RRCS: robotic-assisted rectal cancer surgery, ORCS: open rectal cancer surgery, MD: mean difference, OR: odds ratio; FE: fixed-effects model; RE: random-effects model.
