# Supplementary material for: Robotic-assisted surgery versus open surgery in the treatment of rectal cancer: the current evidence
Source: Sci Rep. 2016 May 27;6:26981. doi: 10.1038/srep26981 (PMC4882598; doi:10.1038/srep26981)
Supplement: Supplementary Tables [file srep26981-s1.pdf]

Robotic-assisted surgery versus open surgery in the treatment of rectal cancer: the current evidence

Guixiang Liao[1#], Yan-Bing Li[2#], Zhihong Zhao[3#], Xianming Li[1*], Haijun Deng[4], Gang Li[5*]

1. Department of Oncology, Shenzhen People's Hospital, Second Clinical Medicine College of Jinan University

2. Department of Hepatobiliary and Pancreatic Surgery, Taihe Hospital, Hubei University of Medicine, Hubei, China

3. Department of Nephrology, The Third Affiliated Hospital of Southern Medical University, Guangzhou, Guangdong

4. Department of General Surgery, Nanfang Hospital of Southern Medical University, Guangzhou, Guangdong

5. Department of Chemoradiation Oncology, The First Affiliated Hospital of Wenzhou Medical University, Wenzhou, China

# Contributed equally

First author: liaoguixiang@163.com

***Corresponding authors:** Xianming Li**, E-mail: chenlhnfy@163.com;** Gang Li, **E-mail: andrewlee0923@163.com**

Supplement tables

Table S1 Quality assessment of Newcastle-Ottawa Scale items

Item	Item details
Selection	1. Assignment for treatment (if yes, one point).<br>2. How representative was the laparoscopic group compared with the general population undergoing rectal resections (if yes, one point; no points if the patients were selected or selection of the group was not described).<br>3. How representative was the open group compared with the general population undergoing rectal resections (if yes, one point; no points if the patients were selected or selection of the group was not described).

Comparability	4. Groups comparable for 1–3 (if yes, two points; one point if one of these three characteristics was not reported, even if there were no other differences between the two groups and other characteristics had been controlled for; no points were assigned if the two groups differed).<br>5. Groups comparable for 4–7 (if yes, two points;<br>one point if one of these four characteristics was not reported, even if there were no other differences between the two groups and other characteristics had been controlled for; no points were assigned if the two groups differed).
Outcome assessment	6. Clearly defined outcome of interest (if yes, one point for information ascertained by medical records or interview; no points if this information was not reported).<br>7. Follow-up equal between the two groups (if yes, one point; no points if follow-up not reported)

Comparability variables: 1 = age, 2 = gender, 3 = ASA, 4 = neoadjuvant/adjuvant therapy, 5 = tumor location, 6 = stage, 7 = procedure

Table S2 Quality assessment of the included non-randomized controlled studies based on the Newcastle-Ottawa Scale

Study	Selections			Comparability		Outcome assessment		Quality score
	1	2	3	4	5	6	7	
Barnajian M[22]	*	*	*	**	*	*		7
Bertani E[23]	*	*	*	**	*	*		7
Desouza AL[24]	*	*	*	**	*	*		7
Gzlle TL[25]	*	*	*	**	**	*	*	9
Kang J[26]	*	*	*	**	**	*	*	9
Kim JC[27]	*	*	*	*	*	*		6
Park JS[28]	*	*	*	*	*	*		6
